# Immunometabolic control of cytokine production by micronutrients in health, aging, and inflammation

**DOI:** 10.3389/fimmu.2026.1781797

**Published:** 2026-05-05

**Authors:** Joyeta Ghosh, Debprasad Chattopadhyay

**Affiliations:** 1 Department of Dietetics and Applied Nutrition, Amity Institute of Applied Sciences (AIAS), Amity University, Kolkata, West Bengal, India; 2 Department of Microbiology & Ethnomedicine, Indian Council of Medical Research (ICMR)-National Institute of Traditional Medicine, Belagavi, India; 3 Department of Virology, Dr Anjali Chatterjee Regional Research Institute (H), Kolkata, India; 4 Department of Microbiology, Vidyasagar University, Midnapore, West Bengal, India

**Keywords:** calcium signalling, cytokines, epigenetics, ferroptosis, immunometabolism, inflammaging, metabolic sensing, micronutrients

## Abstract

Micronutrients serve as critical metabolic sensors and epigenetic regulators that orchestrate cytokine production through multiple overlapping signalling cascades, transcriptional networks, and cellular metabolic states. This comprehensive review synthesizes recent research demonstrating that micronutrient status regulates cytokine biology at five hierarchical levels: (i) nutrient sensing via mTORC1/GCN2 and amino acid sensor networks; (ii) transcriptional control through VDR/RARα-mediated epigenetic remodelling and histone deacetylase inhibition; (iii) redox signalling via SELENOK/selenoprotein-stabilized calcium homeostasis and Nrf2/ARE pathway activation; (iv) Pyroptosis/ferroptosis execution via metallothionein-zinc-caspase axes and NLRP3/GSDMD regulation; and (v) metabolic bioenergetics through NAD+/CD38/SIRT-mediated immune cell differentiation and aging. Recent discoveries establish that vitamin D directly suppresses IL-22 through repressive VDREs independent aryl-hydrocarbon receptor (AhR) signalling, zinc-metallothionein-3 that suppresses non-canonical inflammasome activation via TRIF-IRF3-STAT1 modulation, selenium-dependent SELENOK which stabilizes IP3 receptor-mediated store-operated calcium entry in immune cells, and folate-dependent one-carbon metabolism generating S-adenosyl methionine (SAM) that tunes epigenetic landscapes of cytokine genes. This review compiles the integrated mechanistic frameworks linking micronutrient availability to immunometabolic checkpoints, with implications for nutritional immunotherapy in chronic inflammatory diseases and immune-senescence.

## 1 Introduction: the micronutrient-immunometabolic nexus

The immune system is not merely a static defence apparatus but a highly dynamic metabolic hub that integrates nutritional status with pathogen recognition and inflammatory responses. The recognition that nutrient availability directly governs the fate of immune cell decisions has catalyses a fundamental paradigm shift in immunology: cytokine production is not an all-or-nothing process controlled by transcription factors, but rather an integrated outcome of metabolic status, nutrient availability, epigenetic state, and cellular redox balance (1, 2).

Micronutrients serve as essential cofactors in enzymatic reactions. However, their roles extend far beyond simple catalysis. Recent structural and functional studies demonstrate that micronutrients function as molecular sensors that activate specific intracellular signalling cascades controlling immune cell fate at metabolic checkpoints. The convergence of micronutrient sensing pathways at common effector nodes—particularly the NLRP3 inflammasome, ferroptosis machinery, calcium-calcineurin-NFAT signalling, and NAD+-dependent histone deacetylases explains the broad immunomodulatory effects of seemingly disparate nutrients and accounts for synergistic effects observed in multi-micronutrient supplementation studies (3–6). This review synthesizes recent mechanistic discoveries demonstrating that (i) vitamin D directly inhibits cytokine genes through VDR-mediated transcriptional repression independent of classical immune cell activation pathways; (ii) zinc regulated inflammasome execution through metallothionein-mediated suppression of TRIF-IRF3-STAT1 signalling; (iii) selenium-dependent selenoprotein stabilize calcium homeostasis required for immune cell proliferation; (iv) folate-dependent one-carbon metabolism that generate universal methyl donors controlling epigenetic landscapes of inflammatory genes; and (v) copper and magnesium as essential cofactors in calcium signalling and antioxidant enzyme maturation that control immune cell fate.

In this mini−review, we organize current evidence within a hierarchical “immunometabolic checkpoint” framework that links micronutrient bioavailability to cytokine production across successive control nodes. First, nutrient sensing pathways (mTORC1, GCN2, amino acid and one−carbon sensors) detect fluctuations in micronutrient status and translate these into changes in cellular metabolism. Second, these signals shape epigenetic programming through vitamin D receptor/retinoic acid receptor (VDR/RAR) complexes, folate−dependent S−adenosylmethionine (SAM) generation, and histone deacetylase inhibition, thereby reconfiguring accessibility of cytokine loci. Third, micronutrient−regulated calcium and redox signalling modules (magnesium–TRPM7–STIM–Orai1, selenium−dependent selenoproteins, copper/zinc− dependent antioxidant enzymes) tune NFAT, NF−κB, and Nrf2 activation thresholds. Fourth, these upstream inputs converge on inflammasome and lytic cell death checkpoints (NLRP3 activation, caspase−GSDMD pyroptosis, ferroptosis and iron handling) that determine whether cytokine responses remain adaptive or progress to tissue−damaging inflammation. Fifth, NAD^+^ metabolism and age−associated changes in CD38 and sirtuin activity integrate lifetime nutritional exposures with immunometabolic aging and inflammaging. Finally, we discuss how these checkpoints can be targeted for biomarker−guided, micronutrient−based interventions in chronic inflammatory disease and age−related immune dysfunction.

**Figure 1:**
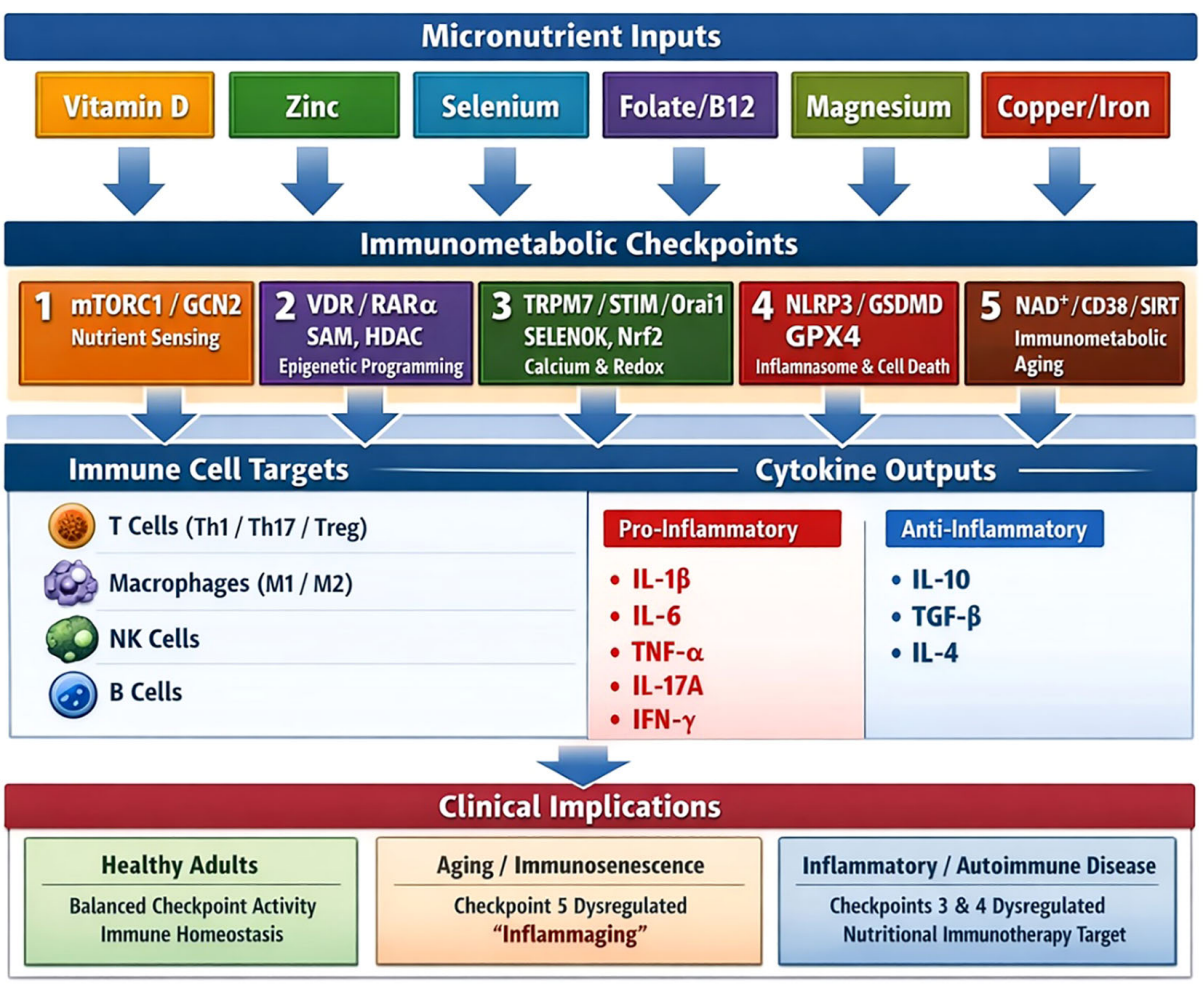
Integrated immunometabolic framework: micronutrient sensing, immune cell regulation, cytokine networks, and clinical outcomes.

**Figure 1** revealed that six key micronutrients (vitamin D, zinc, selenium, folate/B12, magnesium, copper/iron) regulate immune biology through five hierarchical checkpoints: (1) nutrient sensing via mTORC1/GCN2 that controls immune cell fate between pro-inflammatory and regulatory phenotypes; (2) epigenetic reprogramming via VDR/RARα-HDAC complexes and folate-dependent SAM generation that configures cytokine gene accessibility; (3) calcium and redox signalling via TRPM7-STIM-Orai1 and SELENOK-IP3R axes that tune NFAT, NF-κB, and Nrf2 activation thresholds; (4) inflammasome and lytic cell death regulation via zinc-MT3-NLRP3 and iron-GPX4-ferroptosis axes that determine adaptive versus pathological inflammation; and (5) NAD^+^/CD38/SIRT-mediated immunometabolic aging that integrates lifetime nutritional exposures with inflammaging. Downstream effects on T cells, macrophages, NK cells, and B cells produce distinct pro-inflammatory (IL-1β, IL-6, TNF-α, IL-17A, IFN-γ, IL-22) or regulatory (IL-10, TGF-β, IL-4) cytokine profiles, with clinical implications for healthy immune homeostasis, aging-related immune dysfunction, and chronic inflammatory disease (**Figure 1**).

## 2 Nutrient sensing and mTORC1/GCN2 metabolic checkpoints

### 2.1 mTORC1 as an amino acid sensor in immune cell differentiation

This establishes mTORC1 as the primary anabolic checkpoint translating amino acid sufficiency into pro-inflammatory immune cell differentiation, representing the first level of the immunometabolic hierarchy outlined in the Introduction.

The mechanistic target of rapamycin (mTOR) complex 1 (mTORC1) functions as a central hub integrating micronutrient availability with immune cell proliferation and differentiation. Unlike growth factor signalling that activates mTORC1 through a linear pathway (PI3K-Akt-TSC2-Rheb), amino acid sensing operates through parallel, nutrient-specific mechanisms that recognize individual amino acids via dedicated sensor proteins (1, 2). The identification of specific amino acid sensors—including leucine sensor SESN2, arginine sensor SLC38A9, methionine sensor SAMTOR/PRMT1, and threonine sensor TARS2- reveals that cells possess exquisite discrimination for essential amino acids dependent immune responses (1, 2).

Methionine sensing exemplifies the sophistication of nutrient-responsive mechanisms. Unlike directly sensed leucine and arginine, methionine is sensed indirectly through its metabolic product S-adenosylmethionine (SAM). Under methionine-replete conditions, SAM Sensor Upstream Of mTORC1 (SAMTOR) dissociates from GATOR1 complex, allowing PRMT1 (Protein Arginine Methyltransferase-1) to methylate NPR2 Like, GATOR1 Complex Subunit, thereby suppressing GATOR1’s GAP activity and activating mTORC1 (1). Conversely, methionine deprivation causes SAM depletion, SAMTOR-GATOR1 reassociation, and mTORC1 inhibition, thereby coupling one-carbon metabolism directly to protein synthesis machinery (1).

In immune cells, mTORC1 activation promotes pro-inflammatory Th1 and Th17 differentiation by: (i) phosphorylating 4E-BP1 to enhance mRNA translation efficiency; (ii) activating S6K1 to promote ribosome biogenesis and protein synthesis; and (iii) inhibiting IRS1 signalling to suppress regulatory T cell (Treg) differentiation (7). Arginine availability particularly influences this process, as adequate arginine maintains mTORC1-mediated suppression of IRS1 and Akt signalling, preventing Treg differentiation, while arginine deprivation permits IRS1-Akt signalling and promotes Foxp3+ Treg development (1, 7). This nutrient-dependent immune cell fate has profound implications, because amino acid availability during an immune response literally determines whether the response will be inflammatory (high amino acid availability → mTORC1 ON → Th1/Th17) or tolerant (low amino acid availability → mTORC1 OFF → Treg). The significance of this mechanism in immunity lies in its ability to tightly link nutrient availability with immune cell function. By using mTORC1 as a nutrient-sensing hub, immune cells can grow, divide, and differentiate only when essential amino acids such as arginine, leucine, methionine, and threonine are sufficient. This ensures that energetically demanding immune responses—like T-cell activation, clonal expansion, and effector function—occur only under favourable metabolic conditions. Methionine sensing through SAM connects one-carbon metabolism to protein synthesis and epigenetic regulation, which are critical for immune cell fate decisions. Overall, this system prevents ineffective or harmful immune activation during nutrient scarcity while supporting robust and properly regulated immune responses when resources are adequate.

### 2.2 GCN2-ISR pathway: stress-responsive translational control

In contrast to nutrient replete-state signalling, amino acid starvation activates the general control nonderepressible 2 (GCN2) kinase, a eukaryotic initiation factor 2-alpha (eIF2α) kinase that detects uncharged tRNAs accumulation, when cognate amino acids are depleted (1, 2). GCN2 activation leads to eIF2α phosphorylation and integrated stress response (ISR) activation, causing global translation shutdown when paradoxically promoting expression of stress-adaptive genes including activating transcription factor 4 (ATF4) (1).

**Figure 2:**
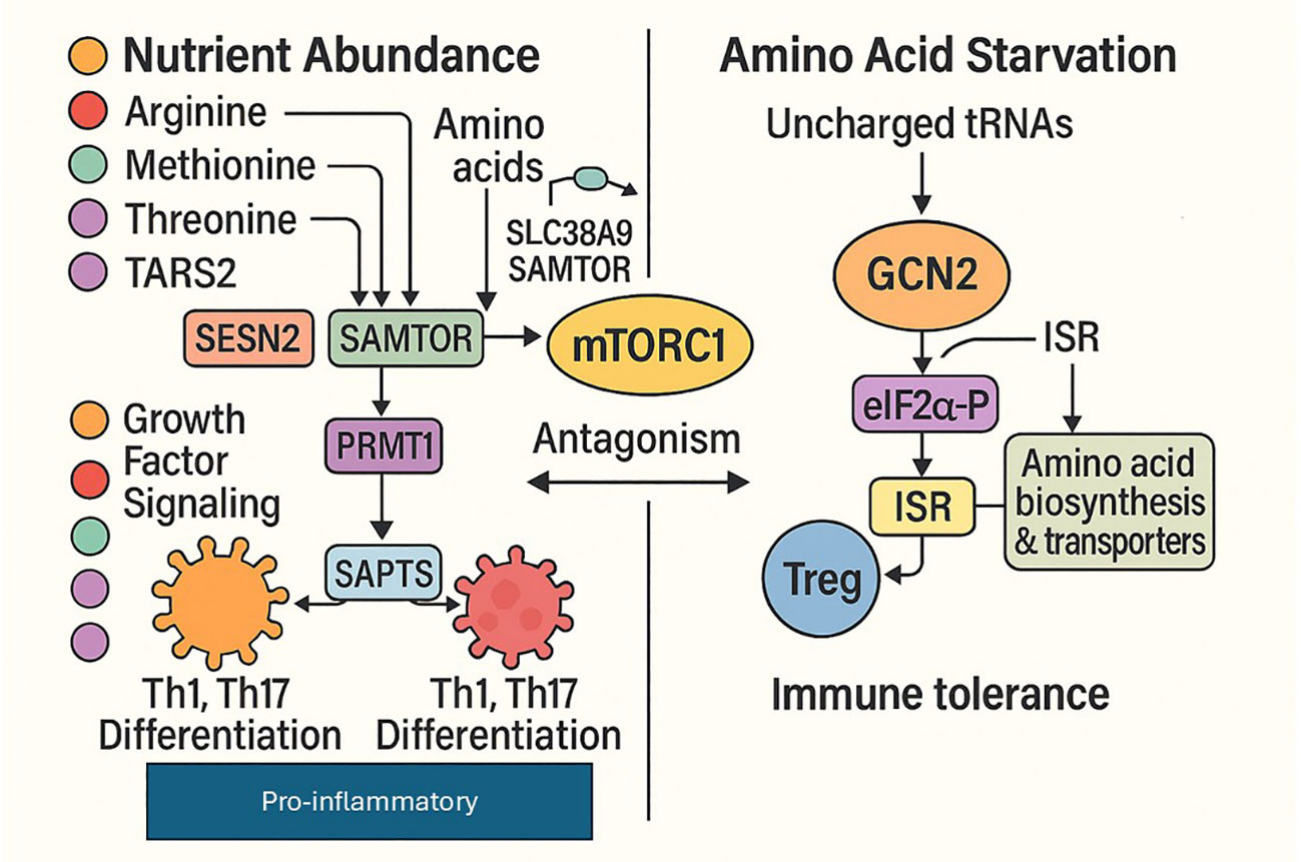
Micronutrient-dependent antagonism between mTORC1 and GCN2 pathways in immune regulation.

**Figure 3:**
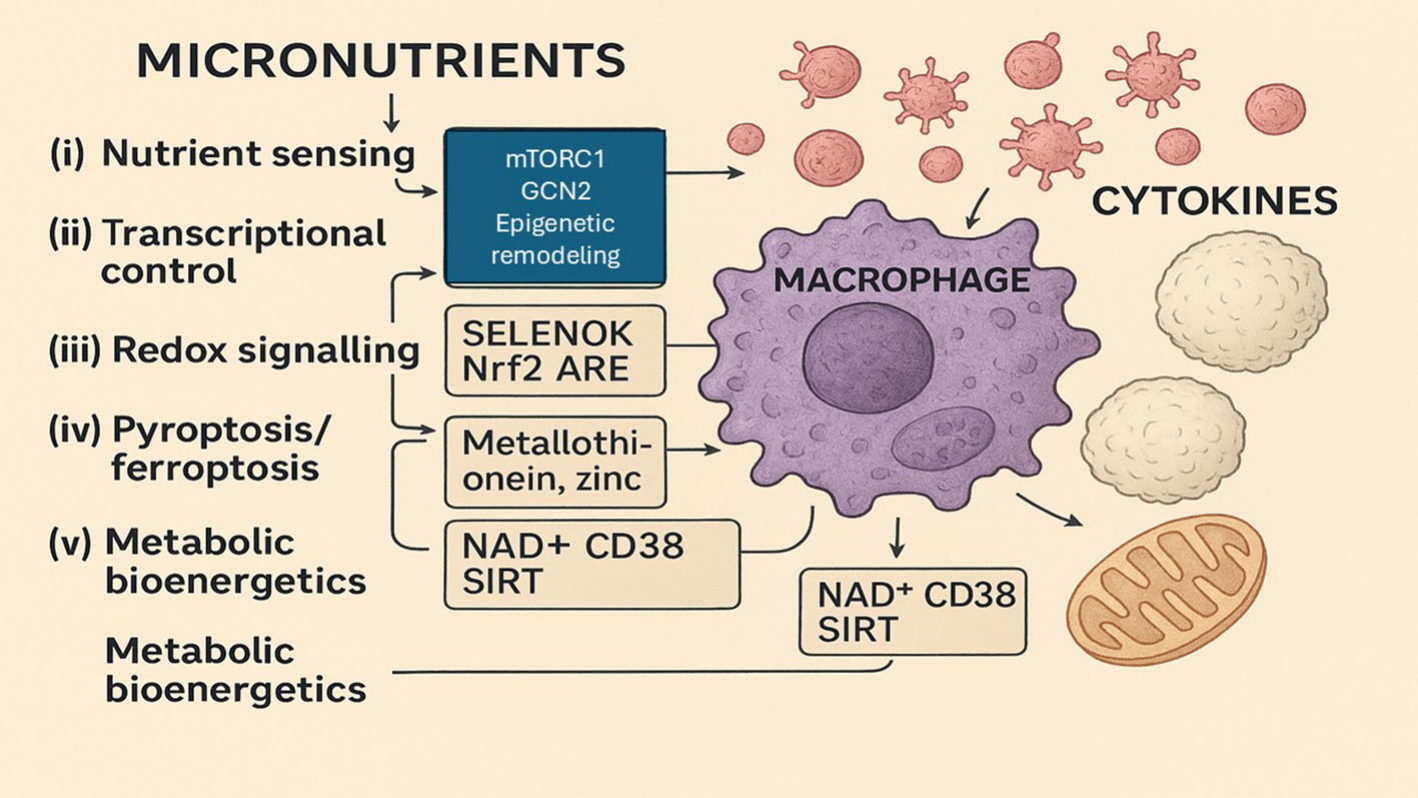
Nutrient sensing and bioenergetic control of macrophage-mediated inflammation.

In immune cells GCN2-ISR activation suppresses pro-inflammatory gene expression through three major mechanisms: (i) ATF4-mediated induction of ATF3, a negative feedback inhibitor of NF-κB; (ii) reduced translation of short-lived pro-inflammatory cytokine mRNAs due to global translation shutdown; and (iii) enhanced expression of amino acid biosynthetic enzymes and nutrient transporter genes, enabling metabolic adaptation to scarcity (1). Notably, GCN2 and mTORC1 operate in reciprocal antagonism: mTORC1 promotes catabolic metabolism and pro-inflammatory differentiation when nutrients are abundant; while GCN2 promotes catabolic autophagy and immune tolerance when nutrients are scarce. This binary switch controlled by micronutrient availability represents a fundamental principle of immunometabolism (1, 2). The significance of this pathway in immunity is its action as a protective brake on immune activation during nutrient scarcity. When amino acids are limited, GCN2 senses this stress and triggers the integrated stress response, reducing overall protein production while selectively activating adaptive genes, such as ATF4. In immune cells, this response dampens excessive inflammation by limiting pro-inflammatory cytokine synthesis, inhibiting NF-κB signalling through ATF3, and promoting metabolic adjustments that help cells to survive low-nutrient conditions. The reciprocal balance between GCN2 and mTORC1 ensures that immune responses are scaled appropriately to nutrient availability—supporting strong inflammatory activity when resources are sufficient and promoting immune tolerance, stress resistance, and survival when nutrients are scarce. This nutrient-dependent switch is a key principle of immunometabolism (**Figures 2**, **3**). Together, mTORC1 and GCN2 form a reciprocal binary switch that collectively translates micronutrient availability into immune cell fate decisions. When amino acids and micronutrients are sufficient, mTORC1 drives pro-inflammatory Th1 and Th17 differentiation; when they are scarce, GCN2 suppresses this response and promotes Treg induction and immune tolerance. This dual-sensor axis constitutes the first immunometabolic checkpoint of the framework introduced in the Introduction, and subsequent sections examine how downstream epigenetic, calcium, redox, and inflammasome nodes further refine the immune outcome.

## 3 Epigenetic programming and one-carbon metabolism

### 3.1 Folate-dependent one-carbon metabolism and SAM generation

Building on the nutrient-sensing mechanisms described above, this section examines how folate availability determines SAM production — the universal methyl donor that configures the epigenetic landscape of cytokine gene loci, constituting the second immunometabolic checkpoint. The folate and methionine cycles are intimately coupled in one-carbon metabolism (OCM), a pathway that generates S-adenosylmethionine (SAM), the universal methyl-donor for epigenetic modifications. Folate undergoes two-step enzymatic reduction via dihydrofolate reductase (DHFR), forming 5,6,7,8-tetrahydrofolate (THF), the active coenzyme carrying single-carbon units at its N5 and N10 positions (8–10).

Methionine adenosyl transferase (MAT) catalyses SAM synthesis from methionine and ATP (consuming 3 high-energy phosphate bonds), making SAM synthesis an extremely energy-intensive process coupled to cellular energy status (10). SAM subsequently acts as a methyl donor in three major epigenetic pathways: (i) DNA methylation via DNA methyltransferases (DNMTs), catalysing 5-methylcytosine (5mC) deposition at CpG dinucleotides; (ii) histone methylation via histone methyltransferases (e.g., EZH2, HMT1/DOT1L), catalysing H3K27me3, H3K4me1, and other repressive/activating marks; and (iii) non-histone protein methylation affecting signalling protein function (10). Critically, SAM consumption by methyltransferases generates S-adenosylhomocysteine (SAH), which is converted back to homocysteine via S-adenosyl homocysteinase (AHCY) (8, 10). The SAM/SAH ratio (ideally >2.0) controls methylation reaction kinetics: when SAM/SAH is low, DNMTs and HMTs operate at reduced capacity, leading to hypomethylation of pro-inflammatory genes (8). Conversely, adequate folate and B_12_ status maintains high SAM/SAH ratios, enabling robust DNA methylation of transposon sequences and oncogenes while simultaneously allowing selective hypomethylation of genes required for immune activation—a dual regulatory capacity essential for appropriate immune homeostasis (10).

In the inflammatory context, folate deficiency creates a “folate trap” where 5,10-methylene THF becomes sequestered and unavailable for recycling, reducing the folate pool available for nucleotide synthesis AND reducing SAM generation (8). This dual deficiency impairs both T and B lymphocyte proliferation (due to reduced dTMP and purine synthesis) and reduces cytokine gene transcription (due to reduced epigenetic methylation capacity). Recent studies demonstrate that adequate folate status enhances histone acetylation and hypomethylation at the Foxp3 promoter region in regulatory T cells, promoting a stable Treg phenotype through ATRA-enhanced histone acetylation combined with reduced repressive H3K27me3 marks (11). The significance of this pathway in immunity lies in its central role in linking micronutrient availability to epigenetic regulation and immune cell function. The folate and methionine cycles together control SAM production, which determines the cell’s capacity for DNA, histone, and protein methylation, the processes essential for regulating immune gene expression. Adequate SAM levels support lymphocyte proliferation by enabling nucleotide synthesis and ensure proper epigenetic silencing or activation of immune-related genes. When folate or vitamin B12 is deficient, reduced SAM generation and a low SAM/SAH ratio impair methylation reactions, leading to dysregulated inflammatory gene expression and weakened T- and B-cell responses. Importantly, balanced one-carbon metabolism allows immune cells to maintain genomic stability, control pro-inflammatory signalling, and promote immune tolerance, as evident in stable regulatory T-cell differentiation. Thus, folate–methionine metabolism is a key metabolic–epigenetic axis underlying immune homeostasis and effective immune regulation.

### 3.2 Vitamin A-RARα and histone deacetylase-mediated epigenetic reprogramming

Building on the folate-SAM-dependent methylation axis described above, all-trans retinoic acid (ATRA) represents a second, convergent epigenetic mechanism operating on the same cytokine gene loci through histone acetylation remodelling rather than methylation, together forming the complete epigenetic programming checkpoint (Level 2 of the immunometabolic framework).

All-trans retinoic acid (ATRA), the active metabolite of vitamin A, represents a prototypical epigenetic modifier that reprograms immune cell fate through histone deacetylase (HDAC) removal and remodelling of histone acetylation landscapes. ATRA binds to retinoic acid receptors (RAR α/β/γ) which heterodimerize with retinoid X receptors (RXR α/β/γ) to form RXR/RAR complexes (12). These complexes bind retinoic acid response elements (RAREs), consensus DNA sequences in the proximal promoters and distal enhancers of target genes, and recruit either coactivator or corepressor complexes controlling gene expression (12).

Mechanistically, ATRA induces the removal of HDAC1, HDAC2, and HDAC3 from RARE-containing regulatory regions in a gene-specific manner (12). For example, HDAC1 and HDAC3 are removed from the Hoxa1 promoter RARE, while HDAC1 alone is removed from the RARβ2 RARE upon ATRA exposure. This HDAC removal permits increased histone H3K27 acetylation (H3K27ac), a canonical activating epigenetic mark associated with active chromatin and transcription. The quantitative magnitude of H3K27ac increase is substantial, as ATRA treatment increases H3K27ac at the Hoxa1 RARE by ~3-fold, at the Cyp26a1 RARE by ~7-8 fold, demonstrating potent epigenetic remodelling (12).

In immune development, ATRA antagonizes the expression of *rorc* (gene encoding RORγt, the master transcriptional regulator of Th17 cells) while simultaneously promoting Foxp3 expression in developing Tregs (13, 14). At the molecular level, ATRA enhances histone acetylation and reduces repressive H3K27me3 marks at the Foxp3 promoter, facilitating transcription of the Foxp3 master transcription factor (11). The mechanisms involve: (i) HDAC removal by ATRA-RARα complexes; (ii) recruitment of histone acetyltransferases (HATs) to Foxp3 regulatory regions; and (iii) demethylation of repressive histone methylation marks by UTX/KDM6A demethylase (11). This epigenetic-mediated Treg enhancement by ATRA reduces pro-inflammatory Th1 and Th17 responses, providing a mechanistic explanation for vitamin A deficiency-induced inflammatory Th17 expansion observed in murine models (13).

### 3.3 B lymphocyte differentiation, antibody production, and regulatory capacity under micronutrient control

B lymphocytes represent a second major immune cell population whose differentiation, immunoglobulin class-switch recombination, and immunoregulatory capacity are directly governed by micronutrient-dependent epigenetic and metabolic mechanisms, complementing the T cell-centric pathways described in Sections 3.1 and 3.2 above.

#### 3.3.1 Folate and B cell clonal expansion

Folate is obligatory for the rapid clonal expansion of B cells during germinal centre reactions because dividing B cells require folate-derived thymidylate (dTMP) and purine nucleotides for DNA replication and for the somatic hypermutation that underlies affinity maturation (9). The folate trap created by folate deficiency, in which 5,10-methylene-THF becomes sequestered and unavailable for nucleotide synthesis, directly impairs both the proliferative magnitude of germinal centre B cell responses and the fidelity of somatic hypermutation at immunoglobulin variable region loci (9). Since the same folate-dependent one-carbon metabolic pathway simultaneously controls SAM generation and epigenetic methylation at immunoglobulin gene loci (described in Section 3.1), folate insufficiency exerts suppressive effects on humoral immunity through dual impairment of B-cell mitotic capacity and epigenetic accessibility at loci undergoing somatic hypermutation.

#### 3.3.2 Vitamin D and class-switch recombination

VDR expression in B cells is inducible and is upregulated upon anti-CD40 plus IL-4 stimulation; at concentrations of 10^−6^ M, 1,25(OH)^2^D^3^ inhibits IgE synthesis by 85.5 ± 9.7% *in vitro* through suppression of ϵ germ-line transcription and NF-κB (p50/p65) protein expression without affecting B cell proliferation, establishing that VDR engagement specifically targets the class-switch recombination machinery rather than B cell viability (15, 16). A low-calcemic VDR agonist replicates this effect, suppressing IgE production by human peripheral B cells by 63.9 ± 5.9% *in vitro* and impairing the IgE response in an OVA/alum-sensitised mouse allergy model *in vivo*, with mechanistic involvement of reduced activation-induced cytidine deaminase (AID) transcript levels (17, 18). These findings establish a VDR-dependent brake on IgE class-switch recombination directly relevant to atopic sensitisation, operating through suppression of ϵ germ-line transcription and AID activity, rather than indirect T cell-mediated cytokine modulation.

#### 3.3.3 Zinc and regulatory B cells

Regulatory B cells (B_regs_), identified as CD19^+^IL-10^+^ B-cells that suppress excessive pro-inflammatory responses through IL-10 secretion, have recently been shown to require adequate zinc for their development from human peripheral blood B cells (19, 20). Zinc-deficient culture conditions significantly decrease B_reg_ generation from purified CD19^+^ B-cells stimulated with CD40L plus TLR9 ligand (CpG-ODN2006), providing the first direct evidence that zinc deficiency impairs B_reg_ differentiation in humans (19, 20). The molecular basis of Breg IL-10 production is CD40-dependent STAT3 phosphorylation: in healthy donors, CD40 ligation induces STAT3 phosphorylation predominantly in CD24^hi^CD38^hi^ Bregs, while this STAT3 activation is specifically defective in the same Breg subset from patients with systemic lupus erythematosus and systemic sclerosis, mechanistically explaining the loss of Breg function in these autoimmune diseases (21, 22). Because zinc modulates STAT3 activity through its role as a cofactor for zinc finger protein assembly and through its indirect effects on JAK kinase activity upstream of STAT3, zinc deficiency by impairing CD40-STAT3-IL-10 signalling would reduce Breg immunoregulatory capacity, shifting the immune balance toward unrestrained Th17 and macrophage-driven inflammation in autoimmune settings.

#### 3.3.4 Iron and B cell mitochondrial function

Iron is essential for mitochondrial electron transport chain activity in the rapidly proliferating B cells of germinal centre reactions, where sustained ATP production is required to support the transcriptional programme of B cell receptor engagement, somatic hypermutation, and plasma cell differentiation. Pro-inflammatory JAK-STAT1 signalling, particularly IFN-γ-driven STAT1 activation, suppresses ferroprotein (SLC40A1) expression in immune cells and promotes intracellular iron retention, creating the permissive conditions for ferroptosis through Fenton -driven lipid peroxidation described in Section 5.3 (23, 24). This bidirectional relationship between iron status and cytokine signalling in B cells, wherein inflammatory cytokines redistribute iron to restrict pathogen access while iron availability shapes the energetic capacity for cytokine gene transcription, mirrors the hepcidin-ferroprotein axis operating in macrophages (Section 5.4), underscoring iron homeostasis as a systemic immunometabolic checkpoint governing multiple lymphocyte lineages (23, 24).

## 4 Calcium signalling and redox-dependent immune activation

Downstream of epigenetic reprogramming, calcium and redox signalling modules act as real-time tuners of NFAT, NF-κB, and Nrf2 activation thresholds, constituting the third immunometabolic checkpoint connecting micronutrient status to cytokine transcription. Rather than focusing on individual channels in isolation, it is now clear that calcium influx and redox balance together function as a central immunometabolic checkpoint for cytokine production. Store−operated calcium entry (SOCE) through STIM–Orai complexes, tuned by magnesium−sensitive TRPM7, sets the activation threshold for calcineurin–NFAT signalling and thus for T−cell and myeloid cytokine responses. In parallel, selenium−dependent selenoproteins and copper/zinc−dependent antioxidant enzymes maintain redox conditions that preserve IP_3_ receptor and SERCA function and prevent oxidative inactivation of calcium handling machinery. Importantly, the quantitative impact of these pathways is cell−type specific: the same perturbation in calcium influx or redox tone can have distinct consequences in naïve versus effector T cells, regulatory T cells, or tissue−resident macrophages. In the following subsections, we therefore use TRPM7−STIM–Orai1 and SELENOK–IP_3_R as exemplars of this broader calcium/redox checkpoint, rather than implying that any single channel alone dictates immune activation.

### 4.1 Magnesium-TRPM7-STIM-Orai1 calcium signalling axis

Calcium signalling represents the critical rate-limiting step in immune cell activation, with calcium entry through store-operated calcium entry (SOCE) channels (STIM1-Orai1 complex) triggering calcineurin-mediated NFAT nuclear translocation and subsequent pro-inflammatory cytokine transcription (5, 6, 25). Recent work has revealed that magnesium controls SOCE through a previously underappreciated mechanism involving Transient Receptor Potential Melastatin TRPM)-7, a calcium/magnesium-permeable ion channel present on the plasma membrane and ER (26). TRPM7 knockout (TRPM7 KO) CD4+ T cells show markedly impaired store-operated calcium entry upon stimulation with thapsigargin, an ER SERCA pump inhibitor that depletes ER Ca2+ stores (26). Mechanistically, TRPM7 senses magnesium status and transduces this information to STIM1-Orai1 complex function: when magnesium is depleted, TRPM7 activity is reduced, leading to diminished refilling of ER calcium stores and impaired NFAT-mediated transcription (26). The functional consequence is dramatic: TRPM7 KO cells show reduced thapsigargin-induced NFATc1 nuclear translocation, with quantitative analysis showing that TRPM7 inhibition, via NS8593 inhibitor) reduces TCR-mediated NFAT translocation in both naïve and total CD4+ T cells (26).

The magnesium coupling to TRPM7-STIM-Orai1-calcineurin-NFAT represents an elegant nutrient-sensing mechanism: when magnesium is abundant (fed state), TRPM7 permits robust calcium signalling → NFAT activation → pro-inflammatory cytokine synthesis. Conversely, magnesium depletion restricts calcium signalling → impaired NFAT activation → reduced inflammatory responses. This explains epidemiological associations between magnesium deficiency and elevated systemic CRP in osteoarthritis and fibromyalgia patients (5). The mechanism also provides a molecular rationale for calcineurin inhibitor-based immunosuppression in autoimmune diseases: cyclosporine and tacrolimus (FK506) directly bind calcineurin, preventing magnesium-calmodulin-calcineurin-mediated NFAT activation regardless of magnesium status (5, 25).

Collectively, these data support the view that magnesium−dependent control of SOCE constitutes a nutrient−sensing gate on NFAT−driven cytokine production, with context−dependent effects across different immune cell subsets.

### 4.2 Selenium-SELENOK-IP3R-Ca2+ homeostasis coupling

While magnesium controls SOCE initiation, selenium-dependent selenoproteins regulate SOCE efficiency through stabilization of IP3 receptor (IP3R) calcium release channels on the ER membrane (27). IP3Rs are ligand-gated calcium release channels respond to inositol 1,4,5-trisphosphate (IP3) which is generated downstream of TCR stimulation via phospholipase C-γ (PLCγ) activation. However, IP3Rs are highly sensitive to redox modification: thiol oxidation on regulatory cysteine residues dramatically reduces the IP3R responsiveness and calcium flux (27).

SELENOK, a specific selenoprotein enriched in immune cells, participates in the ER-associated protein degradation (ERAD) pathway and directly stabilizes IP3Rs through redox-dependent mechanisms (27). Under selenium deprivation, SELENOK protein levels decline, leading to destabilization and degradation of IP3Rs from ER membranes. The functional consequence is profound: SELENOK-deficient immune cells show impaired inositol-1,4,5-trisphosphate (IP3)-triggered ER calcium release and reduced capacity for store-operated calcium entry (SOCE) (27). Importantly, these SELENOK-deficient cells do not show signs of enhanced ER stress or apoptosis, contrary to theoretical prediction, but rather demonstrate selective compromises in immune cell functions that depend on efficient SOCE: proliferation, migration, cytokine secretion, and pathogen protection (27). The broader selenoprotein family also regulates ER-calcium-redox interactions: SELENOM overexpression decreases superoxide production, reduces cytosolic Ca2+ flux, and prevents H_2_O_2_-induced apoptosis in neuronal cells. SELENON, with its N-terminal selenocysteine located in the ER lumen, appears to function as an oxidoreductase reversing H_2_O_2_-induced inhibition of the SERCA2b calcium pump (27). Together, the selenoprotein cluster regulates a coordinated ER-calcium-redox axis: adequate selenium permits maintained IP3R and RyR (ryanodine receptor) stability, preserved SERCA2b pump activity, and efficient SOCE-mediated calcium signalling, while selenium deficiency compromises this system, restricting immune cell function at the calcium homeostasis checkpoint (27).

These findings position SELENOK and related selenoproteins as redox–calcium integrators that link selenium status to the fidelity of calcium−dependent activation in T cells and phagocytes.

### 4.3 NK cell cytotoxicity and micronutrient regulation

Natural killer (NK) cells are innate cytotoxic lymphocytes whose tumour surveillance and antiviral functions are governed by micronutrient-dependent calcium and redox signalling mechanisms that directly intersect with the immunometabolic checkpoints described in Sections 4.1 and 4.2.

#### 4.3.1 Vitamin D and NK cell cytotoxicity

Vitamin D exerts context-dependent modulatory effects on NK cell effector function through VDR-mediated transcriptional regulation. Treatment of peripheral blood NK cells with 1,25(OH)^2^D^3^ (10–100 nM) upregulates inhibitory receptors CD158a and CD158b while reducing the early activation marker CD69 and the degranulation marker CD107a; paradoxically, the same treatment significantly increases polarised perforin granule delivery in conjugated NK cells, demonstrating that vitamin D calibrates activation threshold rather than abrogating cytotoxic capacity (28, 29). Dietary vitamin D^3^ supplementation in a murine model enhanced splenic NK cell activity, restored NKG2D expression suppressed by TGF-β under diabetic conditions, and increased the proportion of mature CD11b^+^ NK cells, effects attributable to VDR-dependent modulation of NK maturation cytokine signalling (30, 31). Vitamin D supplementation in vitamin D-deficient healthy individuals upregulates the NK cell-associated cytotoxicity pathway, increasing expression of five IFN-α subtypes and enhancing NK cell-mediated antibody-dependent cellular cytotoxicity (ADCC), with clinically significant implications for rituximab efficacy in diffuse large B cell lymphoma (32, 33). Collectively, these data establish vitamin D as a checkpoint calibrator of NK cell cytotoxic readiness rather than a simple activator or suppressor.

#### 4.3.2 Zinc and NK cell IFN-γ production

The zinc transporter ZIP8 (SLC39A8) is markedly upregulated in lymphocytes upon activation and transports zinc from lysosomes to the cytosol, where cytoplasmic zinc at 0.8–3.1 μM inhibits calcineurin (CN) phosphatase activity, sustaining phosphorylation of the transcription factor CREB and thereby driving IFN-γ expression (34, 35). siRNA knockdown of ZIP8 in primary human T cells reduces both IFN-γ secretion and perforin release, while ZIP8 overexpression enhances both, establishing a direct mechanistic link between ZIP8-mediated zinc translocation and cytotoxic effector output (34, 35). Because NK cells share the calcineurin-NFAT and calcineurin-CREB signalling architecture with CD8^+^ T cells for IFN-γ production and perforin secretion, zinc deficiency, by impairing ZIP8-mediated zinc translocation, disproportionately reduces NK cell IFN-γ output and cytotoxic granule release, diminishing antiviral and antitumour surveillance.

#### 4.3.3 Magnesium and NK cell NKG2D-dependent cytotoxicity

The most direct evidence linking magnesium to NK cell function comes from patients with X-linked immunodeficiency with Mg^2+^ defect, EBV infection, and neoplasia (XMEN disease), caused by loss-of-function mutations in the magnesium transporter MAGT1. Chaigne-Delalande et al. demonstrated that decreased intracellular free Mg^2+^ causes defective surface expression of the NK activating receptor NKG2D on both NK cells and CD8^+^ T cells, directly impairing cytolytic responses against EBV-infected cells (36, 37). Remarkably, oral magnesium supplementation in XMEN patients restored intracellular free Mg^2+^, rescued NKG2D expression, and concurrently reduced EBV-infected peripheral blood mononuclear cells *in vivo* (36, 37). Consistent with this, extracellular Mg^2+^ promotes LFA-1 conformational activation on cytotoxic lymphocytes by binding to metal-ion-dependent adhesion sites (MIDAS) on CD18 and CD11a, augmenting immunological synapse formation, calcium flux, and specific cytotoxicity (38, 39). Clinically, low serum magnesium correlates with more rapid disease progression and shorter overall survival in patients receiving CAR T-cell therapy and immune checkpoint antibodies (38, 39), identifying serum magnesium as a directly actionable determinant of cytotoxic lymphocyte effector capacity in the oncology setting (39).

#### 4.3.4 Selenium and NK cell redox defence in the tumour microenvironment

NK cells infiltrating solid tumours face intense reactive oxygen species (ROS)-mediated suppression within the tumour microenvironment (TME). IL-15-primed NK cells acquire resistance to oxidative stress through mTOR-dependent activation of the thioredoxin (Trx1) antioxidant system; tumour-infiltrating NK cells from non-small-cell lung cancer (NSCLC) patients express higher Trx1 and surface thiol densities than peripheral blood NK cells, with Trx1 expression predicting NK cell tumour penetrance and potentially immunotherapy response (40, 41). Since thioredoxin reductase-1 (TXNRD1), a selenium-dependent selenoenzyme, is the obligate regenerator of reduced Trx1, selenium sufficiency is a prerequisite for the redox-protective capacity that enables NK cell persistence and cytotoxic function within the oxidative TME (40, 41). Selenium deficiency, by reducing TXNRD1 activity, compromises Trx1 regeneration and impairs NK cell survival and cytotoxic persistence within solid tumours, representing a mechanistic basis through which micronutrient status directly governs innate antitumour surveillance.

## 5 Inflammasome and lytic cell death checkpoints: zinc, magnesium, and iron as micronutrient regulators

The inflammasome–pyroptosis axis is a key effector checkpoint at which micronutrient status can either amplify or restrain inflammatory cytokine release. While the core molecular steps of canonical and non−canonical inflammasome activation are now well defined, our focus here is on how zinc, magnesium and related metals reprogram this pathway by modulating TRIF–IRF3–STAT1 signalling, caspase activation, and GSDMD pore formation. Thus, MT3–zinc complexes, Mg²^+^/Zn²^+^ co−regulation of GSDMD, and the broader metallothionein network are considered as micronutrient−sensitive brakes or amplifiers of IL−1β/IL−18 release and pyroptotic cell death.

### 5.1 Non-canonical inflammasome suppression by MT3-Zn2+ axis

The discovery that zinc-metallothionein-3 (MT3) suppresses the non-canonical (caspase-11-dependent) inflammasome pathway represents a major mechanistic advance, revealing cross-talk between zinc availability and pyroptotic cell death (3). The non-canonical inflammasome is activated when Gram-negative bacteria deliver lipopolysaccharide (LPS) into the cytosol via type 3 secretion systems or outer-membrane vesicles. Cytosolic LPS binds to human caspase-11 or caspase-4/5 (analogous to mouse caspase-11) directly, causing their autoproteolytic activation (3). Activated caspase-11 cleaves gasdermin D (GSDMD), an executioner protein that forms membrane pores permitting pyroptotic cell death and releasing inflammatory mediators. Caspase-11 activation also triggers coordinated activation of the canonical NLRP3 inflammasome through TRIF-dependent signalling, as caspase-11 stimulates TLR adaptor TRIF, which activates interferon regulatory factor 3 (IRF3) and STAT1, leading to expression of inflammasome components (NLRP3, caspase-1, pro-IL-1β) and their subsequent activation (3).

MT3, a zinc-binding metallothionein, antagonizes this cascade at a critical node: zinc imported into macrophages via ZIP8 transporter is sequestered by MT3. The MT3-bound Zn_2_^+^ pool subsequently suppresses TRIF phosphorylation and IRF3 activation—key steps upstream of caspase-11 and canonical inflammasome activation (3). In MT3-knockout macrophages challenged with cytosolic LPS, TRIF phosphorylation is exaggerated, IRF3-STAT1 signalling is hyperactivated, and caspase-11-dependent pyroptosis is dramatically enhanced (3). Conversely, MT3 overexpression or zinc supplementation restrains non-canonical inflammasome activation. Notably, zinc exhibits context-dependent effects on inflammasome pathways: long-term zinc depletion disrupts lysosomal integrity, permitting cathepsin B release and activation of the canonical NLRP3 inflammasome. Conversely, short-term zinc chelation impairs pannexin-1 receptor function and attenuates NLRP3 activation (3). This biphasic zinc response reveals that the relationship between micronutrient status and inflammasome activation is not monotonic but exhibits an optimal window of sufficiency. The significance of this discovery lies in revealing zinc as an active regulator of inflammasome-driven inflammation rather than a passive micronutrient. By identifying MT3 as a zinc-dependent brake on the non-canonical caspase-11 inflammasome pathway, this work uncovers a critical mechanism through which immune cells restrain excessive pyroptotic cell death during Gram-negative bacterial infection. MT3-mediated zinc sequestration limits TRIF–IRF3–STAT1 signalling, thereby dampening both caspase-11 activation and secondary NLRP3 inflammasome amplification. This regulatory axis prevents runaway inflammation while preserving host defence. Importantly, the context-dependent effects of zinc highlight that immune homeostasis depends on maintaining an optimal zinc window, where deficiency or excess can differentially skew inflammasome activation. Together, these findings establish zinc–MT3 signalling as a key immunometabolic checkpoint with therapeutic relevance for inflammatory and infectious diseases characterized by dysregulated pyroptosis.

Together, these findings identify the MT3–zinc axis as a micronutrient−dependent rheostat on caspase−11/NLRP3 inflammasome activity, where zinc sufficiency constrains TRIF–IRF3–STAT1 signalling and thereby limits pathological pyroptosis, whereas zinc imbalance can either exaggerate or blunt inflammasome responses depending on exposure pattern.

### 5.2 Synergistic Mg2+/Zn2+-mediated pyroptosis inhibition via GSDMD

Recent molecular frameworks have elucidated how magnesium and zinc synergistically inhibit pyroptosis by targeting gasdermin D (GSDMD), the executor of pyroptotic pore formation (4). Both classical NLRP3-Caspase-1-GSDMD and non-canonical Caspase-11-GSDMD pathways converge on GSDMD cleavage and activation. The N-terminal domain of GSDMD (GSDMD-NT) released by caspase cleavage oligomerizes in the inner plasma membrane leaflet, forming 14-18 nm diameter pores that permit osmotic cell lysis and release of IL-1β and IL-18 (4).

Mg_2_^+^ and Zn_2_^+^ ions released from metal-organic frameworks (MOFs) synergistically inhibit pyroptosis through coordinated actions: Mg_2_^+^ and Zn_2_^+^ attenuate Gasdermin D (GSDMD) expression at mRNA level and suppress caspase-mediated GSDMD cleavage through mechanisms involving both canonical and non-canonical inflammasome pathways (4). The synergistic effect is substantial: dual Mg/Zn treatment reduces pyroptosis markers more profoundly than either metal alone, with optimal ratios (~20% Mg/Zn) showing maximal suppression of propidium iodide-positive (PI^+^) cells (indicating pyroptotic cell death), decreased NLRP3/Caspase-1 activation, and reduced GSDMD-NT, the activated pore-forming domain (4). The downstream consequences are immunologically significant as Mg/Zn treatment reduces lactate dehydrogenase (LDH) release and IL-1β secretion, preventing the inflammatory cascade amplification that occurs when pyroptotic cells dump inflammatory mediators into the microenvironment (4). In the physiological context, this mechanism suggests that adequate magnesium and zinc availability restrains excessive inflammasome activation during bacterial infections, preventing tissue damage from uncontrolled pyroptosis while preserving microbicidal capacity.

The Mg²^+^/Zn²^+^ co−regulation of GSDMD expression and cleavage suggests that physiological ranges of these trace elements help to confine inflammasome activity to a productive host−defence window, whereas deficiency or supraphysiologic exposure may destabilize this balance and favour excessive lytic cell death.

### 5.3 Ferroptosis and iron metabolism: JAK/STAT/system Xc− axis

While the preceding subsections examined micronutrient control of pyroptotic lytic death, iron governs an entirely distinct but equally consequential cell death program — ferroptosis — through bidirectional crosstalk with pro-inflammatory cytokine signalling, constituting the third arm of this converged inflammasome and lytic death checkpoint.

Iron represents a unique micronutrient whose relationship with cytokine signalling is inherently bidirectional. Pro-inflammatory cytokines including IFN-γ induce ferroptosis through JAK1/2-STAT1 signalling, which suppresses the cystine-glutamate antiporter (System Xc^−^) and depletes glutathione (GSH), the primary cellular antioxidant and cofactor for glutathione peroxidase (GPX)-4 (23, 24). GPX4 is the only enzyme capable of efficiently metabolizing lipid hydroperoxides (LPO), products of lipid peroxidation. When IFN-γ-stimulated STAT1 suppresses System Xc^−^ components (SLC7A11, SLC3A2), intracellular cystine uptake is dramatically reduced, leading to GSH depletion and GPX4 inactivation (23). When GPX4 inactivated, lipid peroxides accumulate and propagate through phospholipid membranes, initiating ferroptotic cell death. IFN-γ signalling simultaneously inhibits iron export through suppression of SLC40A1 (encoding ferroportin 1, FPN1), the sole iron exporter permitting iron egress from cells (23, 24). The dual action, increased iron retention combined with impaired lipid ROS detoxification, creates a permissive environment for ferroptosis as intracellular iron catalyses the Fenton reaction (Fe_2_+ + H_2_O_2_ → Fe_3_+ + OH• + OH^−^), generating hydroxyl radicals that attack polyunsaturated fatty acids and trigger the lipid peroxidation cascade (23).

Although GPX4 and System Xc^-^ are often considered the dominant ferroptosis checkpoints, additional anti−ferroptotic systems are now recognized. Ferroptosis suppressor protein−1 (FSP1) acts independently of GPX4 by regenerating reduced coenzyme Q_10_ (CoQ_10_) at the plasma membrane, thereby preventing propagation of lipid peroxyl radicals. Parallel protective mechanisms include iron sequestration by ferritin and other iron−binding proteins, regulation of labile iron pools via hepcidin–ferroprotein signalling, and remodelling of phospholipid species to reduce the availability of highly peroxidation−prone polyunsaturated fatty acids. These auxiliary systems interact with cytokine−driven JAK–STAT pathways and iron−handling hormones to determine whether inflammatory cues such as IFN−γ result in controlled effector function or in maladaptive ferroptotic tissue injury.

In pathological contexts, this IFN-γ-STAT1-ferroptosis axis contributes to tissue damage in Sjogren’s syndrome (affecting salivary gland epithelial cells), age-related macular degeneration (affecting retinal pigment epithelial cells), and other autoimmune/inflammatory conditions (23, 24). Conversely, STAT3 activation (downstream of IL-10, IL-6 trans-signalling, or other anti-inflammatory signals) upregulates System Xc− and GPX4 expression, conferring ferroptosis resistance and promoting cell survival (23). This STAT1/STAT3 antagonism represents another metabolic checkpoint controlling immune outcome: pro-inflammatory cytokines (driving STAT1) trigger ferroptotic cell death, while anti-inflammatory cytokines (driving STAT3) promote cell survival.

### 5.4 Hepcidin-mediated iron redistribution and IL-6 signalling

The relationship between iron metabolism and cytokine signalling extends to the systemic level through hepcidin, a small cysteine-rich peptide hormone synthesized by hepatocytes and regulated by both IL-6 signalling and local iron status (23, 24). During acute inflammation, IL-6 activates JAK-STAT3 signalling in hepatocytes, inducing hepcidin transcription and synthesis. Hepcidin binds to ferroportin 1 (FPN1) on the plasma membrane of duodenal enterocytes and tissue macrophages, triggering FPN1 internalization and degradation (23, 24).

The physiological consequence is rapid iron sequestration: Hepcidin-mediated FPN1 degradation that prevents iron export from macrophages, leading to iron accumulation in the reticuloendothelial system and systemic hypoferremia—a hallmark of anaemia of chronic inflammation (ACI). This iron sequestration is evolutionarily ancient, likely representing a mechanism to starve invading pathogens of iron, which is essential for their survival and virulence (23, 24). However, chronic hepcidin elevation, driven by persistent IL-6 signalling in autoimmune/inflammatory disease, results in progressive iron sequestration, leading to functional iron deficiency in peripheral tissues despite elevated iron stores in macrophages. The iron redistribution can be reversed by anti-inflammatory cytokines IL-10 and TGF-β that suppress Hepcidin expression and promote iron release from macrophages through upregulation of FPN1 and transferrin receptor-mediated iron uptake into peripheral tissues (23). This bidirectional regulation represents another nutrient-sensing checkpoint: the cytokine profile literally controls iron distribution between immune cells, where iron is needed for ROS generation and antimicrobial activity, and peripheral tissues where iron is needed for oxygen transport and enzymatic function.

## 6 Vitamin D-VDR: transcriptional repression of IL-22 via repressive VDREs

### 6.1 Direct VDR-mediated IL-22 gene silencing

Recent mechanistic studies have fundamentally revised our understanding of vitamin D’s regulation of IL-22, establishing that calcitriol (1,25-dihydroxyvitamin D3) directly silences the *il22* gene through VDR binding to repressive vitamin D response elements (VDREs) in the IL-22 promoter (28, 30, 32). Previous models emphasized indirect effects through suppression of IL-6, which drives IL-22-producing dendritic cells, or through VDR interference with IL-23 signalling. However, new molecular evidence demonstrates that calcitriol-VDR-RXR heterodimers directly bind a VDRE in the *il22* promoter and recruit corepressor complexes, preventing transcription factor access and suppressing IL-22 production (28).

In Th22 cells (CD4^+^ T cells producing IL-22 but not IL-17), calcitriol suppresses *il22* transcription in a VDR-dependent manner as point mutations in the VDR DNA-binding domain abolish the inhibitory effect of calcitriol despite preserved ligand binding and VDR protein levels (28). This VDR-dependent mechanism is independent of effects on other transcriptional regulators of IL-22, including aryl hydrocarbon receptor (AhR) and RORγt. While calcitriol does suppress AhR expression, the direct VDRE-mediated repression of *il22* operates independently and in parallel (28). Structurally, the repressive VDRE in human *il22* promoter exhibits the canonical nucleotide sequence recognized by VDR-RXR heterodimers. Upon calcitriol binding to VDR, the receptor undergoes conformational change promoting interaction with corepressor complexes containing histone deacetylases (HDACs) and chromatin-remodelling enzymes (28). These corepressors maintain repressive histone H3K27me3 marks and restrict histone H3K4ac (activating marks) at the *il22* locus, preventing NF-κB, STAT3, and RORγt transcription factors from accessing their binding sites in the promoter (28).

### 6.2 AhR/IL-22 axis integration in intestinal barrier function

It is known that vitamin D directly suppresses IL-22, while another critical micronutrient tryptophan and its metabolites activate the aryl-hydrocarbon receptor (AhR), which conversely enhances IL-22 production (30, 34). This represents a fundamental physiological integration point as vitamin D and tryptophan metabolites exert opposing effects on IL-22 through distinct transcriptional mechanisms. The AhR/IL-22 axis is critical for intestinal barrier function: activation of AhR in CD4+ T cells, innate lymphoid cells (ILC3), and epithelial cells promotes IL-22 secretion (30). IL-22 acts on epithelial cells via IL-22R1-IL-10R2 heterodimeric receptors, activating JAK-STAT3 signalling that induces production of antimicrobial peptides (RegIII-β, RegIII-γ, lysozyme), tight junction proteins (claudin-1, claudin-15, occludin, ZO-1), and mucins (MUC2) (30, 34). In intestinal inflammation models (DSS-induced colitis), AhR activation through tryptophan metabolites including xanthurenic acid (XANA) and kynurenic acid (KYNA) that increases IL-22 expression, promotes epithelial proliferation and barrier repair (34).

The mechanistic integration is elegant: tryptophan metabolites activate AhR → enhanced IL-22 production → improved epithelial barrier function. In contrast, vitamin D acts to restrict excessive IL-22 responses through direct transcriptional repression. The physiological balance between these nutrient-mediated pathways likely determines intestinal barrier integrity by adequate tryptophan and AhR signalling (through diet-derived metabolites) support barrier function, while excessive vitamin D or D-signalling in genetically susceptible individuals may shift the balance toward barrier disruption through IL-22 suppression. This antagonism provides a molecular explanation for the observation that vitamin D supplementation sometimes exacerbates inflammatory bowel disease symptoms in subset of patients (32).

## 7 Copper-metallothionein-SOD1: antioxidant enzyme maturation and redox signalling

Copper homeostasis is classically discussed in terms of mitochondrial respiration and antioxidant enzyme maturation, but from an immunological perspective its key relevance lies in how copper availability shapes redox−sensitive signalling and lymphocyte proliferation. Copper chaperone networks that deliver copper to SOD1 and other cuproenzymes determine the capacity of immune cells to detoxify superoxide and maintain thiol–disulfide equilibrium, thereby influencing activation thresholds for NF−κB, AP−1, and other redox−regulated transcription factors that control cytokine production.

### 7.1 Copper chaperone for SOD1 and thiol-disulfide equilibrium

Copper functions as an essential cofactor for dozens of enzymes critical to immunity, including cytochrome C oxidase (respiration), lysyl oxidase (collagen cross-linking), and critically, superoxide dismutase 1 (SOD1) (36, 38, 40). However, free copper is highly toxic as it can generate reactive oxygen species through Fenton-like reactions. Cells have therefore, evolved sophisticated copper trafficking machinery including the copper importer CTR1 and the intracellular copper chaperone copper chaperone (CCS) for SOD1 (36, 40). CCS binds copper(I) through its N-terminal MxCxxC motif (where x = any amino acid) and transfers copper to SOD1 through a transient heterodimer complex (36, 40). The CCS-SOD1 heterodimer formation is exquisitely controlled as zinc-bound SOD1 readily forms heterodimers with CCS, while zinc-deficient SOD1 either fails to form heterodimers or forms “frozen” heterodimers that prevent catalytic copper transfer (36). This zinc-copper cooperation is essential: SOD1 requires both copper (catalytic centre for superoxide dismutation) and zinc (structural cofactor maintaining protein fold and copper coordination geometry) for activity (36).

Recent structural and mechanistic studies of pathogenic R163W CCS mutation, identified in an infant with fatal neurological abnormalities, revealed that this single nucleotide substitution eliminates zinc binding in CCS and paradoxically converts CCS from a copper chaperone to a copper scavenger (40). The R163W CCS accumulates in an aggregation-prone state and exhibits remarkably high affinity binding for copper in its alternative Sod1-like domain (D2), effectively sequestering copper and preventing its transfer to SOD1 (40). The functional consequence is profound as SOD1 loses its copper cofactor and becomes catalytically inactive despite normal synthesis, leading to accumulation of superoxide anion (O2•−) and increased oxidative stress.

Thus, although structural work on CCS–SOD1 complexes is highly informative, the immunological implication is that disturbances in copper and zinc co−coordination can inactivate SOD1, increase superoxide burden, and lower the threshold for oxidative activation of inflammatory signalling pathways.

### 7.2 Copper regulation of immune cell T-cell proliferation and IL-2 production

Copper availability directly regulates immune cell function through control of copper-dependent enzyme activity. Copper deficiency reduces lymphocyte proliferation and impairs IL-2 production in response to mitogenic stimulation (38). While the precise copper-sensing mechanisms in immune cells remain incompletely defined. Evidence suggests that copper regulates IL-2 signalling through control of copper-dependent signalling proteins and redox-sensitive transcription factors (38). In cancer immunotherapy, copper depletion achieved through inhibition of copper transporters (SLC31A1) or through copper chelators (e.g., trientine) shows promise in overcoming cancer immune evasion and enhancing anti-PD-1 checkpoint inhibitor efficacy (38). The mechanisms involve: (i) selective targeting of cancer stem cells depend on copper-dependent proliferation pathways; (ii) inhibition of the histone methyltransferase EZH2 (polycomb repressive complex 2 component) whose activity requires copper; and (iii) potential restoration of anti-tumour T-cell responses through enhanced IL-2 signalling (38). A recent clinical trial combining copper depletion (trientine) with anti-PD-1 therapy in advanced oral squamous cell carcinoma reported an objective response rate of 57.8% and 12-month overall survival of 80.0%, suggesting that micronutrient-targeted immunotherapy may overcome immunotherapy resistance (38).

Taken together, these observations indicate that copper is not only required for canonical antioxidant enzyme activity but also modulates IL−2 signalling, T−cell proliferation, and anti−tumour immunity, positioning copper handling as a potential immunometabolic target in both chronic inflammation and cancer immunotherapy.

## 8 NAD^+^ metabolism and CD38-SIRT axis: aging-related immune dysfunction

Pharmacological strategies to raise NAD^+^ levels using precursors such as nicotinamide riboside or nicotinamide mononucleotide have shown heterogeneous outcomes in early−phase clinical and preclinical studies, with some reports of improved metabolic and inflammatory markers and others showing minimal benefit. Moreover, because many tumour cells and highly proliferative immune cells also depend on NAD^+^−driven metabolism, there is a theoretical risk that chronic NAD^+^ augmentation could support tumour growth, alter immune surveillance, or favour expansion of pathogenic immune subsets in certain contexts. These observations underscore the need for carefully stratified, disease−specific trials and combinatorial approaches (e.g. modulation of CD38 or sirtuin activity) rather than assuming that NAD^+^ elevation is universally beneficial in aging and inflammation.

### 8.1 CD38 as NAD+-consuming enzyme in aging

The discovery that CD38, an ectoenzyme with both NAD glycohydrolase and ADP-ribose cyclase activities, is dramatically upregulated with aging has fundamentally revised understanding of aging-related metabolic decline (16, 17, 19). CD38 cleaves NAD^+^ into nicotinamide (NAM) and adenosine diphosphate ribose (ADPR), with ADPR additionally functioning as a second messenger that triggers calcium release through ryanodine receptor activation (16). CD38 expression increases ~2.5-fold in adipose tissue of older individuals and shows elevated activity (2-3-fold higher) in cancer patients, demonstrating that CD38 is a bona-fide aging biomarker (16). The physiological consequence of elevated CD38 in aging is profound as aged mice exhibit approximately 50% reduction in NAD^+^ levels compared to young mice, while CD38 knockout aged mice maintain youthful NAD^+^ levels and resist metabolic dysfunction associated with high-fat diet feeding (hepatic lipidosis, glucose intolerance) (17). Mechanistic studies revealed that increased CD38 expression is the primary driver of age-related NAD^+^ decline, not impaired NAD^+^ synthesis (16, 17). While other NAD^+^-consuming enzymes (PARPs, SIRTs) remain relatively stable with age, CD38 activity increases dramatically (16).

The evolutionary logic underlying CD38 upregulation in aging remains unclear but may involve enhanced calcium signalling in aged immune cells for inflammatory amplification. However, the pathological consequence is NAD^+^ depletion and mitochondrial dysfunction: CD38 knockout or CD38 inhibition (78c inhibitor) restores NAD^+^ levels and improves glucose tolerance, skeletal muscle function, cardiac function, and exercise capacity in aged mice (17). These findings establish NAD^+^ availability as a critical determinant of health span in aging and suggest that micronutrient-dependent NAD^+^ metabolism represents a fundamental checkpoint in immunosenescence.

### 8.2 SIRT-mediated epigenetic control of inflammatory gene expression

The sirtuins (SIRT1-7) are NAD^+^-dependent histone and protein deacetylases that regulate immune cell function through multiple mechanisms. SIRT1, the prototypical sirtuin, deacetylates histone H3 and H4 in pro-inflammatory gene promoters, removing activating histone acetylation marks and suppressing expression of TNF-α, IL-6, IL-1β (16). In immune cells, SIRT1 activation promotes differentiation toward Tregs while suppressing pro-inflammatory Th1 differentiation (16). Notably, the NAD^+^-SIRT axis couples energy status to immune cell fate decisions: during fasting or metabolic stress (conditions elevating NAD^+^/NADH ratio), SIRT1 activity increases, promoting Treg differentiation and anti-inflammatory responses. Conversely, in nutrient replete states (conditions lowering NAD^+/^NADH), SIRT1 activity decreases, permitting pro-inflammatory gene expression. This nutrient-dependent immune cell fate provides an evolutionary rationale as during famine, organisms suppress inflammatory responses to conserve energy and resources, while during nutrient abundance, robust inflammatory responses to infection are prioritized (16). With aging and CD38-mediated NAD^+^ depletion, this regulatory system becomes dysregulated: SIRT activity chronically declines, leading to sustained hyperacetylation of inflammatory gene promoters and constitutive pro-inflammatory cytokine expression or inflammaging (16, 17). Micronutrient supplementation that supports NAD^+^ synthesis pathways (supporting NAMPT-nicotinamide phosphoribosyl-transferase activity through adequate b3/niacin status) or that inhibits CD38, represents an emerging strategy to restore SIRT-mediated epigenetic suppression of inflammatory genes and counter immunosenescence (16, 17, 19).

## 9 Clinical translation and emerging therapeutic applications

### 9.1 Biomarker-guided micronutrient supplementation in chronic inflammation

The mechanistic frameworks detailed above provide scientific rationale for moving beyond generic multivitamin supplementation toward personalized, biomarker-guided micronutrient protocols. Blood micronutrient status containing zinc, selenium, vitamin D, folate, B_12_, copper should be assessed in patients with chronic inflammatory conditions. Additionally, functional biomarkers reflecting micronutrient-dependent enzyme activities (SOD activity, plasma homocysteine indicating methionine remethylation capacity, NAD^+^/NADH ratio) provide insights into nutritional status beyond simple serum nutrient levels.

Recent clinical data demonstrate the efficacy of this approach: personalized micronutrient supplementation guided by baseline biomarkers of inflammation, micronutrient insufficiency, and immune dysfunction produced clinically significant improvements over 12 weeks (21). All participant groups achieved reductions in high-sensitivity CRP (3.5-4.6 mg/L to 1.9-2.9 mg/L, representing 33-46% reductions), with 100% achieving CRP <3 mg/L, the threshold for low-grade chronic inflammation (21). Simultaneously, ferritin (inflammation marker and iron status), homocysteine (one-carbon metabolism capacity), and anti-TPO antibodies (autoimmune activation) all improved significantly (21).

### 9.2 Tryptophan metabolite-based immunotherapy in IBD

The mechanistic discoveries regarding tryptophan-AhR-IL-22-barrier function have catalysed development of novel therapeutics targeting the tryptophan metabolism pathway in inflammatory bowel disease. Rather than supplementing tryptophan directly (which is abundantly available from dietary proteins), new approaches involve enhancing production of specific tryptophan metabolites including xanthurenic acid (XANA) and kynurenic acid (KYNA) through enzymatic rewiring strategies (34). In DSS-induced colitis mouse models, treatment with enzyme-producing strategies that generate XANA and KYNA from dietary tryptophan shows superior efficacy compared to conventional anti-inflammatory therapies, with protective effects mediated through AhR-IL-22 axis activation and enhanced epithelial proliferation/barrier repair (34). The mechanisms involve: (i) direct AhR activation in epithelial cells enhancing barrier function gene expression; (ii) AhR activation in CD4^+^ T cells and ILC3 enhancing IL-22 production; (iii) IL-22-mediated induction of antimicrobial peptides (RegIII-β, RegIII-γ) and tight junction proteins (34). The metabolic effects also include enhanced glycolysis in Th17 cells (supporting pro-inflammatory differentiation when needed for barrier defence) through AhR-dependent mechanisms (34).

### 9.3 NK cell and B cell targeting in adjuvant nutritional immunotherapy

The mechanistic insights described above regarding micronutrient regulation of NK cells and B lymphocytes have direct translational implications across three clinically relevant contexts. Vaccination: Micronutrient repletion, particularly vitamin D, zinc and folate, represents an underutilised strategy to improve vaccine-induced humoral (B cell) and NK cell-mediated cellular immune responses, especially in elderly and immunocompromised populations. The age-related decline in germinal centre B cell activity and NK cell cytotoxicity that underlies poor vaccine immunogenicity in older adults is mechanistically linked to the deficiencies in these same micronutrients documented in this population. Pre-vaccination micronutrient assessment and targeted repletion may therefore offer a low-cost adjuvant strategy to improve vaccine efficacy in vulnerable groups. Cancer immunotherapy: Magnesium and selenium sufficiency are required to support NK cell immunological synapse formation and DNAM-1-mediated tumour cell recognition. These micronutrient-dependent NK cell functions are complementary to checkpoint blockade immunotherapy (anti-PD-1/PD-L1), which releases T cell-intrinsic suppression but does not restore NK cell effector capacity. Monitoring and correcting magnesium and selenium status in patients receiving checkpoint inhibitors may therefore represent a simple approach to preserve the NK cell arm of anti-tumour immunity. Autoimmune and inflammatory disease: Zinc-driven expansion of IL-10-producing regulatory B cells (Bregs) offers a precision immunomodulatory approach in inflammatory diseases characterised by deficient Breg activity, including systemic lupus erythematosus and inflammatory bowel disease. Combined with the established roles of vitamin D in suppressing AID-dependent class switching and folate in supporting Treg differentiation, these findings collectively support micronutrient-guided immune rebalancing as a complement to biological disease-modifying therapies (42, 43).

## 10 Conclusion

Micronutrients function as master regulators of cytokine biology through multiple hierarchical mechanisms linking nutrient sensing (mTORC1/GCN2) to metabolic fate decisions (NAD^+^-SIRT axis) and encompassing epigenetic remodelling, calcium signalling, and redox control. The convergence of multiple micronutrients on common effector nodes (NLRP3 inflammasome, ferroptosis machinery, NFAT signalling) explains synergistic effects of balanced micronutrient status and provides mechanistic rationale for nutritional immunotherapy.

The discovery that vitamin D directly silences IL-22 through VDREs, zinc-metallothionein-3 suppresses the non-canonical inflammasome through TRIF-IRF3 modulation, and folate-dependent SAM generation controls epigenetic. The landscapes of inflammatory genes represent fundamental advances in understanding how nutritional status determines immune outcomes. These mechanisms connect ancient evolutionary nutrient-sensing systems to modern chronic inflammatory diseases, providing scientific foundation for precision nutritional immunotherapy.

Based on updated understanding the future therapeutic strategies should include: (1) personalized, biomarker-guided micronutrient supplementation rather than generic multivitamins; (2) integrate micronutrient status assessment with immunophenotyping and microbiota profiling; (3) target specific immunometabolic checkpoints (mTORC1 in pro-inflammatory diseases, CD38 in immunosenescence); and (4) leverage nutrient-responsive epigenetic mechanisms to reprogram inflammatory gene expression patterns. Recognition that micronutrients are not simply nutritional requirements but active nutri-pharmaceutical agents controlling immune cell fate decisions will reshape clinical practice and generate novel therapeutic approaches to chronic inflammatory disease.
